# Antioxidant Flavonoid Diosmetin Is Cardioprotective in a Rat Model of Myocardial Infarction Induced by Beta 1-Adrenergic Receptors Activation

**DOI:** 10.3390/cimb45060297

**Published:** 2023-05-29

**Authors:** Taseer Ahmad, Taous Khan, Annet Kirabo, Abdul Jabbar Shah

**Affiliations:** 1 Department of Pharmacy, Abbottabad Campus, COMSATS University Islamabad, University Road, Abbottabad 22060, Pakistan; 2 Laboratory of Cardiovascular Research and Integrative Pharmacology, College of Pharmacy, University of Sargodha, University Road, Sargodha 40100, Pakistan; 3 Division of Clinical Pharmacology, Department of Medicine, Vanderbilt University Medical Center, Nashville, TN 37232, USA

**Keywords:** flavonoid diosmetin, antioxidant, cardioprotective, ECG, cardiac biomarkers, histopathology

## Abstract

Myocardial infarction (MI) is a common and life-threatening manifestation of ischemic heart diseases (IHD). The most important risk factor for MI is hypertension. Natural products from medicinal plants have gained considerable attention globally due to their preventive and therapeutic effects. Flavonoids have been found to be efficacious in ischemic heart diseases (IHD) by alleviating oxidative stress and beta-1 adrenergic activation, but the mechanistic link is not clear. We hypothesized that antioxidant flavonoid diosmetin is cardioprotective in a rat model of MI induced by beta 1-adrenergic receptor activation. To test this hypothesis, we evaluated the cardioprotective potential of diosmetin on isoproterenol-induced MI in rats by performing lead II electrocardiography (ECG), cardiac biomarkers including troponin I (cTnI) and creatinine phosphokinase (CPK), CK-myocardial band, (CK-MB), lactate dehydrogenase (LDH), alanine aminotransferase (ALT), and aspartate aminotranferase (AST) by using biolyzer 100, as well as histopathological analysis. We found that diosmetin (1 and 3 mg/kg) attenuated isoproterenol-induced elevation in the T-wave and deep Q-wave on the ECG, as well as heart-to-body weight ratio and infarction size. In addition, pretreatment with diosmetin attenuated the isoproterenol-induced increase in serum troponin I. These results demonstrate that flavonoid diosmetin may provide therapeutic benefit in myocardial infarction.

## 1. Introduction

Myocardial infarction (MI) is life-threatening and the most common manifestation of ischemic heart disease (IHD), also called coronary artery disease (CAD) [1]. According to world health organization (WHO), IHD is one of the main cause of deaths worldwide, responsible for more than nine million deaths in 2016 [2]. Almost 39% of deaths in developing countries under the age of 70 years are due to IHD and MI [3]. IHD leads to many pathological changes, including increase in oxidative stress, inflammation, necrosis, and lipid peroxidation (LPO), and decline in the nitric oxide (NO) signaling pathway [4]. The oxidative stress and uncontrolled free radicals mainly effect the myocardium of the heart by initiating the inflammatory pathway [5].The activation of this pathological pathway also initiates the lipid peroxidative products, which, in response, suppress the level of natural antioxidants, catalase (CAT), glutathione peroxidase (GPx), and superoxide dismutase (SOD), respectively. The initiation of this pathway plays major role in the development of myocardial damage in MI [6]. Atherosclerosis is also reported to actively participate in pathological changes during MI [7]. That is why atherosclerosis is considered to be the most common cause of MI. Hypertension, hyperlipidemia, diabetes, and smoking are major risk factors for atherosclerosis. In addition, inflammation, inhibition of nitric oxide synthase (NOS) activity, and decline in nitric oxide pathway lead to endothelial dysfunction, which initiates atherosclerotic plaque formation [8,9,10].

Furthermore, the expression of calcium channels [11], intracellular calcium load [12], potassium channels inhibition [10], and the increase in the level of cTnI, CPK, CK-MB, LDH, ALT, and AST are important biomarkers [13,14,15]. CTn is the gold standard to assess MI [16].

The cardioprotection mechanism(s) include all those approaches, which contribute to the prevention of pathological changes in myocardium [17]. Therapeutic approaches include drugs such as vasodilator to decrease vascular resistance, adrenergic antagonist to inhibit overactive sympathetic activity, and calcium channel antagonist. Unfortunately, some undesirable effects are also related to this therapeutic approach, such as cluster headaches with nitroglycerine, withdrawal effects and sexual dysfunction with β-adrenergic antagonist, and reflex tachycardia and hypotension with calcium channel blockers (CCBs) [18].

As a substitute approach to search for alternative drugs, many phytochemicals from medicinal plant sources have been reported as demonstrating cardioprotective properties [19,20]. Natural products including flavonoids have been reported for their protective response against IHD [21,22]. Some studies show that flavonoids reduce the risk of hypertension and CVD [23,24]. Other examples of flavonoids include hesperetin, luteolin, and naringenin, to mention but a few [24]. Flavonoids have also been reported to exert multiple biological effects, including cardioprotective [24], antioxidant [25], and anti-inflammatory [26,27].

**Figure 1:**
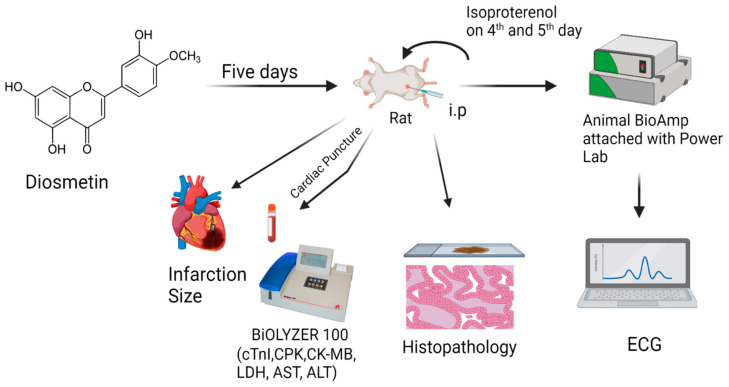
Research protocol of cardioprotective activity of diosmetin against. The isoproterenol induced myocardial infarction.

Concerning diosmetin (5,7,3′-trihydroxy-4′-methoxyflavone), Figure 1 is a monomethoxyflavone, a flavonoid that can be found in lemon peel and citrus fruit [28,29]. Lemon (*Citrus limon* L.), olives (*Olea europaea* L.), and *Rosmarinus officinalis* L. are sources of diosmetin with reported anti-oxidant and cardioprotective activities [30,31,32]. Diosmetin is one of the important phytochemical constituent in these plants [30,31,32,33,34,35]. Diosmetin is reported in anti-oxidant [36,37] hypolipidemic [38], anti-inflammatory [39], antiproliferative, and pro-apoptotic [40] studies. Previous studies have proven the relationship between the mentioned activities and management of MI. Some preliminary studies by Manivannan et al., 2015 and Mo et al., 2020 [41,42] have reported the potential cardioprotective responses of diosmetin, but there is no detailed study available related to the diosmetin effect on beta 1-adrenergic receptors, cardiac biomarkers, ECG pattern, myocardial infarction size and histopathology. Hence, based on clinically reported activities of plant sources and the mentioned pharmacological effects of antioxidant flavonoid diosmetin, the present study was designed.

## 2. Methods

### 2.1. Animals Used

Preferably male adult rats (200–250 g) were used in this study and were kept at the animal house at standard conditions. The protocol of this study was approved (17-06-2013 video notification EC/PHM/07-2013/CUI/ATD) by the Ethical Committee (EC), Department of Pharmacy, COMSATS University Islamabad, Abbottabad campus, Pakistan.

### 2.2. Experimental Protocol

#### Diosmetin Response against the MI Induced by β_1_-Adrenergic Receptors Activation

As cited in the literature [43,44,45], the MI was induced by activating beta 1-adrenergic receptors using a synthetic catecholamine, isoproterenol (ISO), at maximal doses (80 mg/kg). The ISO was administered subcutaneously at a gap of 24 h. Before ISO administration, we administered diosmetin (1 and 3 mg/kg) for 4 days, and on the 5th and 6th day, we administered both ISO and diosmetin. The 1% DMSO was used as a solvent of diosmetin. Simultaneously, atenolol at 1 mg/kg was used as a positive standard to compare the response of diosmetin. The details of the five groups (n = 6) are shown in Table 1.

The body weights of rats were measured before and at the end of the experiment. Then anesthesia was induced by using thiopental at a dose of 80–60 mg/kg.

After that, the rats were ready to measure the ECG by using lead II ECG, consisting of needle electrodes connected to BioAmp and PowerLab (AD Instruments, Sydney, Australia). The ECG was analyzed by using Lab Chart 7. To analyze the different MI biomarkers, cTnI, CPK, CK-MB, LDH, AST, and ALT in serum, blood samples were also collected. The standard assay kits were used for analysis and were carried out through Biolyzer 100. To compare the anatomical changes in the hearts of different groups, the heart weight of all the experimental rats were noted [15,46]. The isolated hearts of different groups of rats were then preserved in the 10% formalin solution for histopathological study. For staining H and E, stains were used and heart tissue were mainly investigated under light microscope (200×) for changes in the branched appearance of cardiac muscle fibers, denoting inflammation which is characterized by edema and ultimately leads to necrosis [43,47].

### 2.3. Statistical Significance Analysis

The GraphPad Prism software version 9.5.1 was used to analyze the data. One-way ANOVA followed by Tukey’s test was applied, to compare the statistical significance of results. The data was declared statistically significant with *p* less than 0.05.

## 3. Results

### 3.1. Electrocardiograph (ECG) Analysis of Different Groups of Experimental Rats

**Figure 2:**
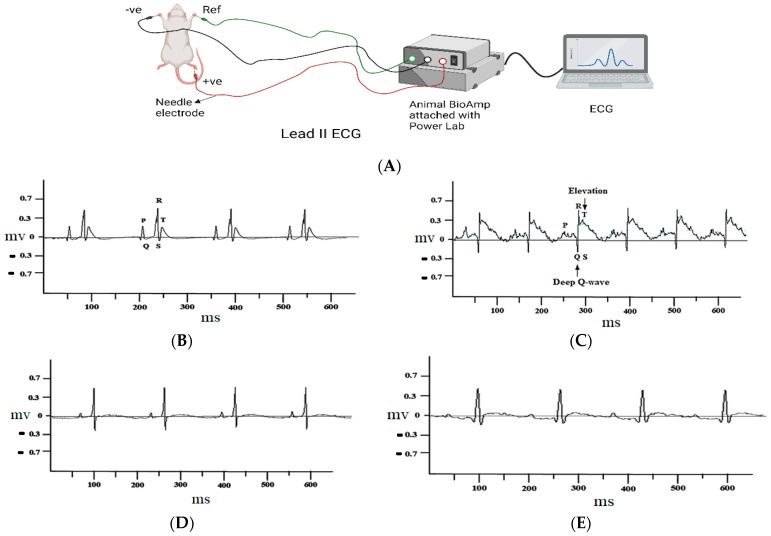

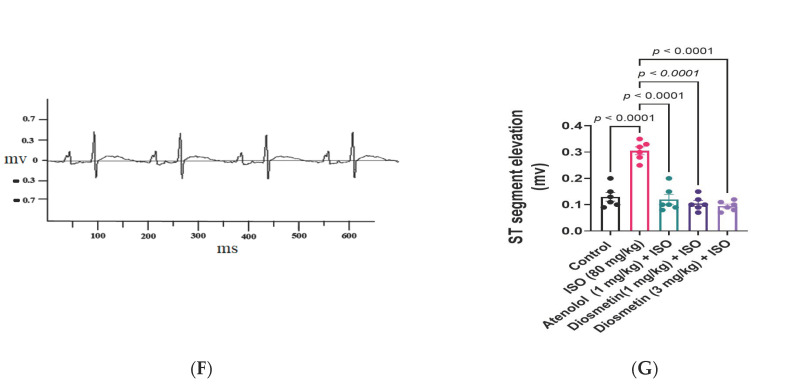
Typical electrocardiogram (ECG) tracings. (**A**) Lead II ECG setup; (**B**) control group, (**C**) isoproterenol group; (**D**) atenolol (1 mg/kg); (**E**) diosmetin, 1 mg; (**F**) diosmetin, 3 mg/kg; and (**G**) ST-segments elevation comparison in tracings (**C**–**G**). Analysis by 1-way ANOVA with a Tukey multiple comparisons post hoc test. n = 6, expressed as mean ± SEM.

In vehicle (1% DMSO) control group, no significant change in the ECG pattern was observed (Figure 2B). ISO-injected rats showed a significant change in the ST-segment elevation, Q wave, unequal PR interval and tachycardia (Figure 2C). Interestingly, in the atenolol- and diosmetin-treated groups, the changes in the ECG patterns were prevented significantly (Figure 2D–F). The significant different in ST-elevation among different groups is shown in Figure 2D.

### 3.2. Heart to Body Weight Ratio

**Figure 3:**
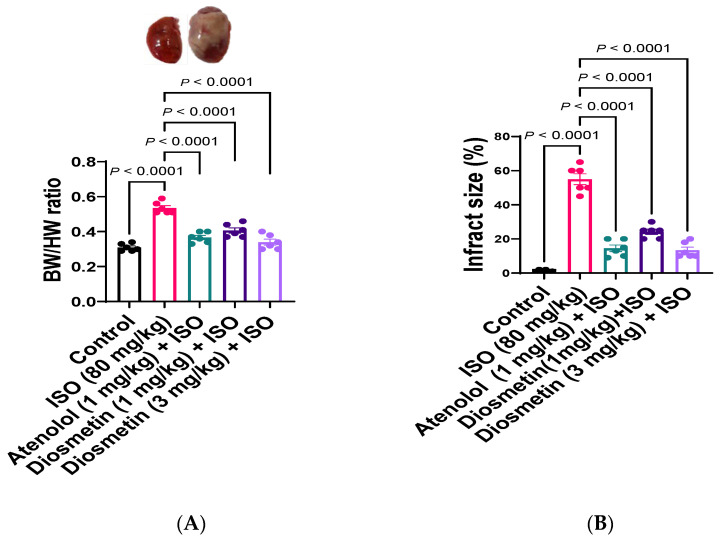
Heart to body weight ratio and infarction size. (**A**) Body weight/heart weight (BW/HW) ratio of diosmetin- and ISO-treated groups in comparison to the control group. (**B**) The % infarction size of the heart in control and experimental rats. Results are expressed as the mean ± SEM of six determinations. Analysis by 1-way ANOVA with a Tukey multiple comparisons post hoc test.

No significant change in the body weight (BW) of all experimental rats were observed at the end of experiment. In ISO-alone-injected rats, a significant change in the ratio of heart to BW was witnessed, in comparison to the control group. The atenolol (1 mg/kg) and diosmetin (1 and 3 mg/kg) pre- and co-treated ISO group significantly prevented changes in heart to BW ratio in comparison to the ISO-alone-injected group (Figure 3A).

### 3.3. Anatomical and Histopathological Analysis

In the ISO-alone-administered group, a significant anatomical change in the heart shape was observed in compared to control group rats. This remodeling of rat heart shape also confirms that MI is successfully induced. The white or pale grey area in cardiac tissues is identified as an infarct area. This infarct area was significantly reduced in atenolol (15%)- and diosmetin 1 mg/kg (24%)- and 3 mg/kg (13%)-administered groups as compared to isoproterenol (55%), as shown in Figure 3B.

### 3.4. Protective Response of Diosmetin against Alteration in Cardiac Biomarkers

**Figure 4:**
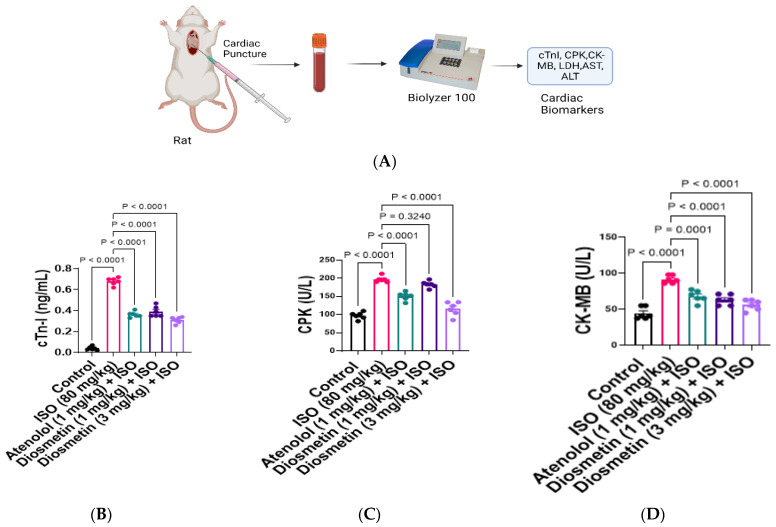

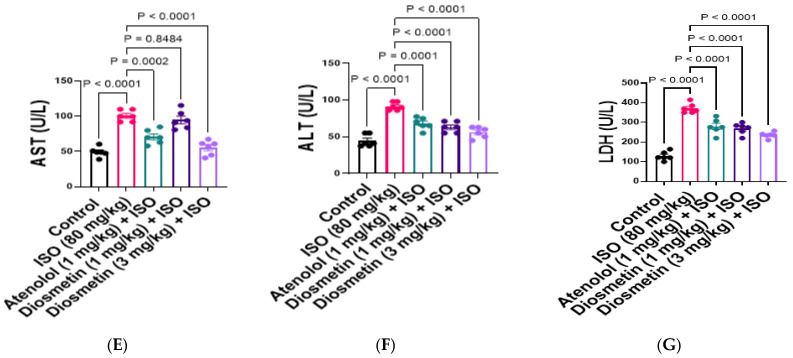
Levels of cardiac biomarkers in the serum of control and treated groups. (**A**) Experimental design of cardiac markers’ estimation in blood serum. Effect of diosmetin and atenolol on cardiac markers’ enzymes in serum of isoproterenol (ISO)-induced ischemic rats; (**B**) troponin I (cTnI); (**C**) creatine kinase-MB (CK-MB); (**D**) creatine phosphokinase (CPK); (**E**) lactate dehydrogenase (LDH); (**F**) alanine transaminase (ALT); and (**G**) aspartate transaminase (AST). Results are expressed as Mean ± SEM (n = 6). Analysis by 1-way ANOVA with a Tukey multiple comparisons post hoc test.

In comparison to control, in ISO-alone-administered rats, a significant escalation was witnessed in the serum levels of cardiac markers including, cTnI, CPK, CK-MB, LDH, AST, and ALT (Figure 4B–G). Atenolol at 1 mg/kg significantly prevented the pathological changes induced by ISO. Moreover, diosmetin (1 and 3 mg/kg) significantly restored the alteration in biomarkers, as compared to the ISO-alone-treated group.

### 3.5. Diosmetin Prevent Histopathological Changes Induced by ISO

**Figure 5:**
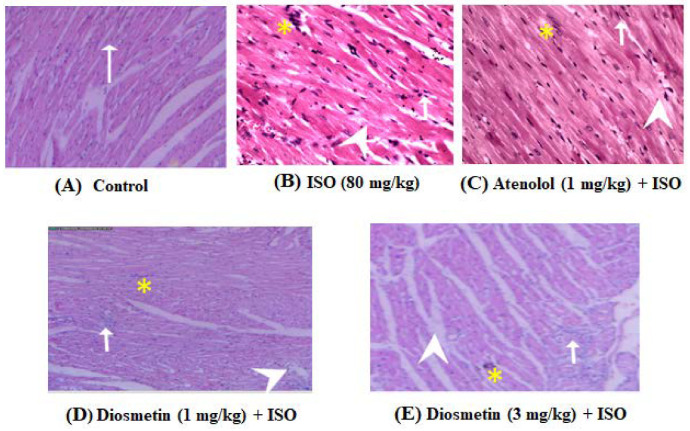

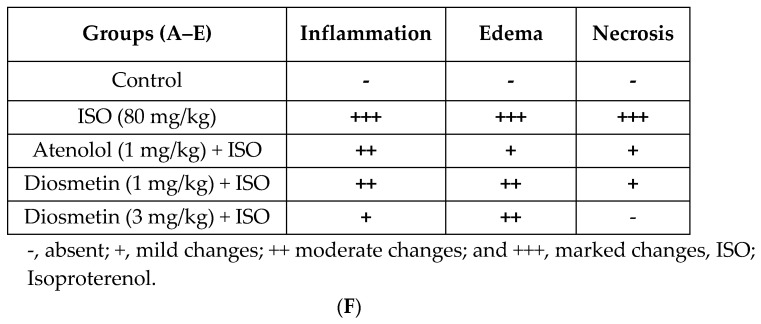
Effect of diosmetin on histopathological changes. Photomicrographs of histopathological examination of heart tissue (H and E, 200×) of the control and ISO-treated experimental rats. The heart section from (**A**) shows normal cardiomyocytes indicated by arrow; the heart section from (**B**) shows marked inflammation indicated by the arrow, edema indicated by the arrow head, and confluent necrosis indicated by the asterix; the heart section from (**C**) revealed moderate reduction in inflammation, edema and necrosis; and the heart section from (**D**,**E**) shows normal cardiac fibers with maximal reduction of inflammation edema and without any necrosis. (**F**) Qualitative analysis of different groups showing the preventive effects of diosmetin and atenolol on the degree of histological changes in comparison to ISO-alone-treated group.

The control group’s myocardium histology shows normal striation with a branched appearance, as shown in Figure 5A, while ISO administration alone at high doses leads to inflammation followed by edema and then ultimately necrosis (Figure 5B). The section of heart tissue sections from atenolol- (1 mg/kg) (Figure 5C) and diosmetin (1 and 3 mg/kg) (Figure 5D,E)-injected groups showed a significant decrease in inflammation, edema and necrosis and the myocardium histology was relatively well protected, with the lowest indication of necrosis compared to the ISO-alone-injected group, as shown in Figure 5F.

## 4. Discussion

The plant sources of diosmetin are reported for antioxidant and cardioprotective activities [31,32]. We tested the protective response of diosmetin in an ISO-induced MI rat model to confirm its cardioprotective activity. ISO is a non-selective β-adrenergic receptor agonist. Stimulation of β_1_-adrenergic receptor activation leads to myocardial contractility. High doses of ISO lead to cardiac toxicity. Maximal stimulation of the β_1_-adrenergic signaling pathway leads to the activation of L-type Ca^2+^ channels. This influx of calcium, in response, releases more calcium from the intracellular store(s) and increase oxidative stress [46,47]. The major finding of this study is that diosmetin significantly inhibited the pathological changes induced by submaximal administration of isoproterenol. Administration of a 80 mg/kg of ISO produced acute stress in the cardiac muscles of SD rats, resulting in necrosis, ST interval elevation, pathological Q-wave, which, looking deep in the ECG tracing and pathological remodeling of the heart shape, is very similar to those patients who are effected by MI [48,49]. Persistently increased heart rate leads to oxygen demand and speeds up myocardial necrosis [43].

The changes in the ECG pattern are considered one of the key and primary clinical markers to diagnose MI. Its accurate elucidation is commonly the basis for instant therapeutic interventions and different analyses for diagnosis. Variations in the ECG events, such as elevation of the ST-event and the deep Q-wave, may be due to the pathological changes in plasma membrane, which lead to abnormal electrical signaling in myocardium [50,51].

Pre-administration of different doses of diosmetin and atenolol (1 mg/kg) significantly restrained ISO-induced changes in ECG patterns, elevation in the ST-event, and deep Q-wave, proposing membrane protective effects. In comparison, diosmetin highly significantly restored the ECG pattern at a 3 mg/kg dose. The cardioprotective response of diosmetin was further confirmed through assessing the cardiac biomarkers.

Cardiac muscles contain copious amounts of diagnostic biomarkers; after MI, the plasma membrane leakage leads to the release of different markers into systemic circulation [52]. So, to confirm clinically the incidence of MI, validation of the cardiac injury markers is vital [53].

**Figure 6:**
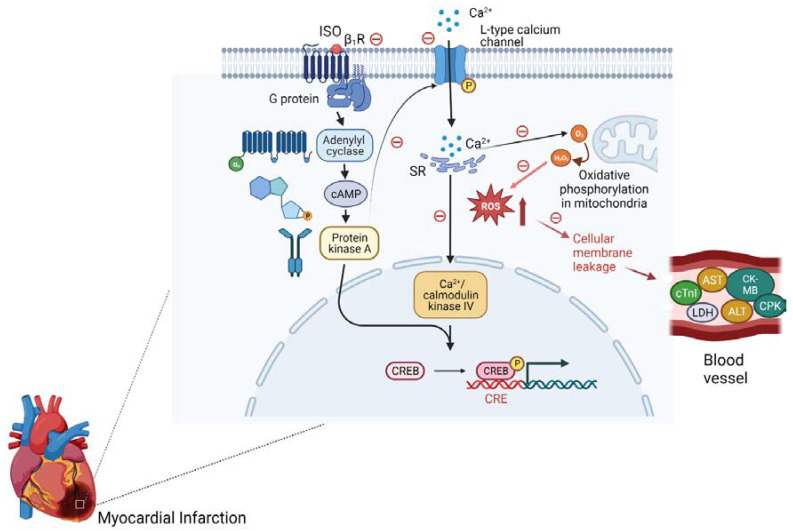
Schematic diagram showing the cardioprotective response “θ” of diosmetin against the isoproterenol (ISO)-induced myocardial infarction. Key: ALT, alanine transaminase; AST, aspartate transferase; β1R, β1 adrenergic receptors; CPK, Creatine phosphokinase; CK-MB, Creatine Kinase MB; cTnI, cardiac troponin I; LDH, lactate dehydrogenase; ROS, reactive oxygen species; SR, sarcoplasmic reticulum.

The potent cardiac muscle contraction by ISO initiates the release of biomarker enzymes in response to myocardial damage, hypoxia, and this subsequently leads to necrosis (Figure 6). Moreover, significant changes in the heart-to-body-weight ratio, with abnormal histopathology of cardiac tissue, confirm ISO-induced MI, which is comparable with previously reported studies [54,55,56,57]. Pretreatment with diosmetin and atenolol and co-administration of ISO significantly prevented the change in heart-to-body-wight ration and infarct size when compared to only-ISO-injected rats. The minimum infarct area was observed in animals pretreated with diosmetin (13%) at 3 mg/kg.

Furthermore, diosmetin also significantly decreased the serum levels of cTnI. A significant decline in the cTnI serum level was detected with pretreatment of diosmetin (55%) at 3 mg/kg. cTnI is a gold standard for diagnosing MI [58]. High serum levels of CPK have usually been considered to be an indirect biomarker of cardiac muscle injury. A significant decline in the CPK level was witnessed with 3 mg/kg diosmetin (41%), while diosmetin also significantly decreased (54%) the serum level of CK-MB. One more important marker which was released in serum after MI is CK-MB [59,60]). Another biomarker which indicates damage and inflammation in cardiac tissues is LDH [61]. The diosmetin (3 mg/kg) also significantly decreased (38%) the serum level of LDH. In physiological conditions, the concentration of AST and ALT is low in the serum. However, post-MI pathological changes also include high levels of markers AST and ALT in serum [62]. The diosmetin also significantly decreases (40%, 46%) the serum concentration of AST and ALT in comparison to ISO-alone-administered rats.

Moreover, a significant infarct zone in rat myocardium was observed with ISO-alone treatment, which is characterized by edema, inflammation, necrosis, and the cardiac muscle fibers’ irregular appearance. It is reported that diosmetin inhibits inflammation by resisting expression of interleukin6 (IL-6), phospho-nuclear factor-kappaB (p-NF-κB), and phospho-c-Jun N-terminal kinases (p-JNK), respectively [63]. ISO at submaximal dose is reported to deplete ATP levels, cause Ca^2+^ overload, increase oxygen demand, and generation of free radicals [64]. However, pre-administration of diosmetin and atenolol counter the pathological changes initiated by ISO injection. Previous studies reported that diosmetin exhibited a significant free-radical-hunting response [36,37]. The diosmetin structure (Figure 1) contains multiple hydroxyl (OH) groups, which also elucidates its capability to prevent free radical damage [65].

The most significant effects of diosmetin were noticed at 3 mg/kg. Plants containing diosmetin have shown increases in levels of SOD, GPX, and CAT [66,67,68]. In addition, diosmetin is known to restore these enzymes in myocardium and inhibit MDA formation [38]. MDA is also considered an important indicator of lipid peroxidation [69]. Lipid peroxidation plays a major role in the generation of oxidative stress [70]. This additional antioxidant effect of diosmetin might complement its cardioprotective potential. The studies by Si et al., 2020 and Mo et al., 2020 [42,71] also reported diosmetin as a cardioprotective agent, decreasing oxidative stress and inducing myocardial apoptosis. Our studies shows that diosmetin blocks the L-type calcium channel and inhibits the release of intracellular calcium in isolated porcine coronary artery and rat aorta [72,73]. So, the cardioprotective response of diosmetin might be due to calcium-channels-blocking activity. The limitation of this study is that more biological markers at molecular level can be traced, such as expression of Bcl-2, Bax, Caspase-3, and proinflammatory factors such as TNF-α in the heart of normal and ISO-administered rats.

Briefly, the data suggest that the pre-administration of diosmetin significantly prevented the pathological changes induced by ISO. The diosmetin significantly preserved the myocardial damage, which is confirmed by the ECG pattern, and the presence of cardiac biomarkers in serum within the range. Further studies would provide more insight into the molecular aspects of these mechanism(s), including the protein expression of Bcl-2, Bax, and Caspase-3 in the myocardium of control and treated rats.

## Figures and Tables

**Table 1:** Distribution of experiment rats in different groups for diosmetin cardioprotective study.

S.NO.	Groups	Procedure
1	Control	■ 1 to 5% DMSO (1 mL/kg, i.p., 5 days); ■ Normal saline (1 mL/kg, s.c.) on 4th and 5th day.
2	ISO	■ 1 to 5% DMSO (1 mL/kg) for 5 consecutive days ISO, 80 mg/kg, s.c. on 4th and 5th day, at 24 h gap.
3	Atenolol + ISO	■ This group received atenolol (1 mg/kg i.p) for 5 consecutive days and ISO at 4th and 5th days.
4	Diosmetin + ISO	■ Diosmetin at 1 and 3 mg/kg, i.p. for 5 days and also ISO at 4th and 5th.

## Data Availability

Data is available on request.
